# Supplementary material for: The Effect of Scale Insects on Growth Parameters of cv. Chardonnay and cv. Sauvignon Blanc Grapevines Grown in a Greenhouse
Source: Int J Mol Sci. 2023 Jan 12;24(2):1544. doi: 10.3390/ijms24021544 (PMC9866550; doi:10.3390/ijms24021544)
Supplement: Supplementary file 1 [file ijms-24-01544-s001.zip › Table S2Relative VOC concentractions_26Nov2022_updated.pdf]

Table S2. Individual samples of leaves used in the HS-SPME-GC/MS analysis showing the calculated relative VOC concentrations and the mean VOC concentrations in ng g^{-1} against their corresponding authentic 10 μg/L standard. Each batch sample ID are leaves from an individual plant. Stdev = standard deviation, and RSD = relative standard deviation.

Sample	Infected_SB	Infected_SB	Infected_SB	Infected_SB	Infected_SB	Infected_SB	Infected_SB	Infected_SB	Infected_SB	**Mean Conc. (ng/g)**	**Stdev**	**%RSD**
**GC/MS Batch Sample ID**	2	3	13	25	26	27	39	40	41			
**Sample Mass (g)**	0.523	0.5365	0.5035	0.5006	0.4999	0.5003	0.5046	0.4994	0.5035			
**Hexanal**	228.0	233.8	412.8	171.8	177.1	159.6	165.4	375.8	241.9	**240.7**	**92.9**	**38.6**
**2-Hexenal, (E)-**	1121.1	875.6	1056.0	926.7	1277.2	825.4	1047.6	906.7	1202.1	**1026.5**	**154.7**	**15.1**
**5-Hepten-2-one, 6-methyl-**	118.5	135.9	159.4	100.3	127.6	129.3	120.9	92.5	121.7	**122.9**	**19.4**	**15.8**
**o-Cymene**	1.5	1.9	2.2	2.4	2.2	2.0	3.4	2.4	4.6	**2.5**	**0.9**	**37.1**
**1-Hexanol, 2-ethyl-**	8.0	6.2	3.1	7.1	7.4	5.2	6.2	5.6	7.2	**6.2**	**1.5**	**23.7**
**Benzyl alcohol**	24.2	7.4	11.5	9.1	8.7	18.1	12.1	11.4	8.0	**12.3**	**5.5**	**44.8**
**p-Cymenene**	4.4	7.1	9.6	9.7	9.2	7.0	17.3	7.6	15.8	**9.7**	**4.2**	**43.3**
**Phenylethyl Alcohol**	27.8	27.1	17.8	35.0	32.1	29.6	31.9	32.6	28.7	**29.2**	**5.0**	**17.0**
**2,6-Nonadienal, (E,Z)-**	12.5	14.7	26.5	11.4	12.2	9.5	13.2	9.7	18.4	**14.2**	**5.3**	**37.4**
**Methyl salicylate**	0.99	0.66	0.41	0.29	0.91	1.47	0.29	1.18	0.31	**0.7**	**0.4**	**60.3**
**Decanal**	11.95	12.02	34.70	14.73	22.69	20.35	16.91	32.89	15.83	**20.2**	**8.5**	**41.8**
**1-Cyclohexene-1-carboxaldehyde, 2,6,6-trimethyl-**	3.30	3.57	4.95	2.56	4.07	2.47	3.45	2.22	5.55	**3.6**	**1.1**	**31.6**
**ß-Citral**	1.66	1.50	0.77	1.45	1.55	1.55	1.23	1.62	1.86	**1.5**	**0.3**	**21.2**
**α-Citral**	5.48	5.53	3.22	6.04	5.61	7.06	5.53	6.51	7.31	**5.8**	**1.2**	**20.5**
**Propanoic acid, 2-methyl-, 3-hydroxy-2,2,4-trimethylpentyl ester**	82.45	86.71	103.59	92.19	110.71	93.84	107.67	115.38	108.27	**100.1**	**11.6**	**11.6**
**Tetradecane**	2.04	1.83	1.52	2.48	2.51	2.80	0.98	1.68	0.99	**1.9**	**0.7**	**34.9**
**α-Ionone**	2.41	3.03	4.25	1.63	3.55	1.91	1.24	2.26	2.20	**2.5**	**1.0**	**38.2**
**α,ß-Dihydropseudoionone**	32.60	32.58	38.84	30.84	32.86	34.95	30.16	30.25	27.63	**32.3**	**3.2**	**10.0**
**9-epi-ß-Caryophyllene**	4.35	3.77	2.55	3.19	3.56	3.70	0.93	1.80	0.94	**2.8**	**1.3**	**46.1**
**trans-ß-Ionone**	5.03	5.75	6.14	3.51	6.14	4.23	3.50	4.32	5.67	**4.9**	**1.1**	**21.7**
**ß-Ionon-5,6-epoxide**	19.07	20.67	28.52	13.27	21.21	15.33	14.36	16.33	20.74	**18.8**	**4.7**	**24.9**
**Pentadecane**	1.79	1.72	1.99	1.65	1.89	2.03	0.94	1.44	0.90	**1.6**	**0.4**	**26.6**
**Dihydro-actinidiolide**	10.24	10.21	14.00	6.71	8.47	6.16	6.25	6.37	9.20	**8.6**	**2.6**	**30.3**
**Hexadecane**	5.35	5.53	6.35	5.03	6.58	6.48	4.56	6.24	4.50	**5.6**	**0.8**	**14.6**
**Heptadecane**	7.35	9.48	12.08	6.95	9.23	9.13	8.52	9.93	7.90	**9.0**	**1.5**	**17.2**
**Octadecane**	2.26	3.16	5.79	2.03	4.11	3.77	3.95	4.04	3.42	**3.6**	**1.1**	**30.8**
**Farnesyl acetone**	5.79	5.62	5.12	6.84	4.73	7.04	5.09	3.20	4.09	**5.3**	**1.2**	**23.2**
**Eicosane**	0.23	0.44	1.84	0.17	0.48	0.53	0.56	0.44	0.51	**0.6**	**0.5**	**85.3**
**Heneicosane**	0.03	0.02	0.26	0.02	0.15	0.17	0.18	0.17	0.19	**0.1**	**0.1**	**66.3**
**Hexadecanoic acid, butyl ester**	13.33	17.81	23.20	6.82	9.60	10.89	17.63	6.85	6.95	**12.6**	**5.9**	**46.7**
**Docosane**	0.00	0.01	0.15	0.00	0.09	0.10	0.16	0.14	0.09	**0.1**	**0.1**	**76.2**
**Tricosane**	0.00	0.00	0.13	0.08	0.19	0.16	0.28	0.25	0.18	**0.1**	**0.1**	**70.5**

Sample	SB	SB	SB	SB	SB	SB	SB	SB	SB	**Mean Conc. (ng/g)**	**Stdev**	**%RSD**
**GC/MS Batch Sample ID**	4	5	6	19	20	21	33	34	35			
**Mass (g)**	0.4998	0.5079	0.5087	0.5011	0.5022	0.4995	0.501	0.5034	0.5019			
**Hexanal**	228.3	245.0	205.2	230.7	243.2	202.1	210.7	235.4	223.4	**224.9**	**15.8**	**7.0**
**2-Hexenal, (E)-**	1246.2	996.9	971.6	916.5	1331.4	1160.8	1298.1	1270.6	1186.8	**1153.2**	**154.0**	**13.4**
**5-Hepten-2-one, 6-methyl-**	125.2	120.4	161.0	178.9	94.5	99.7	91.2	132.8	100.0	**122.6**	**30.8**	**25.1**
**o-Cymene**	1.3	1.8	2.3	2.6	2.7	2.6	2.2	2.5	3.0	**2.3**	**0.5**	**22.1**
**1-Hexanol, 2-ethyl-**	8.0	5.6	6.1	6.4	7.0	7.7	11.0	6.8	9.1	**7.5**	**1.7**	**22.4**
**Benzyl alcohol**	23.6	25.5	21.1	20.9	70.1	7.1	12.9	56.2	10.4	**27.5**	**21.4**	**77.7**
**p-Cymenene**	3.0	3.4	9.4	10.3	16.3	8.7	8.9	13.6	10.3	**9.3**	**4.2**	**45.3**
**Phenylethyl Alcohol**	32.3	27.9	28.1	27.2	28.8	33.4	38.6	31.4	31.7	**31.1**	**3.6**	**11.5**
**2,6-Nonadienal, (E,Z)-**	11.2	10.5	18.9	20.0	22.4	12.9	12.5	16.7	16.9	**15.8**	**4.2**	**26.6**
**Methyl salicylate**	8.95	4.18	0.32	0.98	0.79	0.26	1.40	1.68	0.59	**2.1**	**2.8**	**132.7**
**Decanal**	19.76	24.98	12.93	19.32	10.53	13.17	20.64	21.56	20.42	**18.1**	**4.8**	**26.4**
**1-Cyclohexene-1-carboxaldehyde, 2,6,6-trimethyl-**	2.95	2.78	4.07	4.62	3.12	3.11	2.53	3.59	4.33	**3.5**	**0.7**	**21.2**
**ß-Citral**	1.36	0.89	0.96	1.31	1.00	1.67	1.48	1.40	2.22	**1.4**	**0.4**	**30.1**
**α-Citral**	4.70	3.92	4.70	5.34	4.95	5.83	5.22	4.95	6.51	**5.1**	**0.7**	**14.4**
**Propanoic acid, 2-methyl-, 3-hydroxy-2,2,4-trimethylpentyl ester**	58.32	75.12	87.50	117.68	113.60	86.73	89.21	117.35	120.21	**96.2**	**22.0**	**22.9**
**Tetradecane**	2.40	2.35	1.54	2.56	2.07	2.52	2.49	2.06	2.43	**2.3**	**0.3**	**14.4**
**α-Ionone**	3.53	2.81	2.27	2.57	1.63	2.54	1.85	2.04	3.36	**2.5**	**0.6**	**25.8**
**α,ß-Dihydropseudoionone**	32.35	33.19	37.27	35.11	31.39	31.47	28.88	32.04	29.49	**32.4**	**2.6**	**8.1**
**9-epi-ß-Caryophyllene**	4.27	3.72	3.47	2.95	1.63	3.18	3.43	2.21	3.21	**3.1**	**0.8**	**25.3**
**trans-ß-Ionone**	5.29	3.53	5.64	6.29	4.62	4.95	3.95	4.69	6.75	**5.1**	**1.0**	**20.5**
**ß-Ionon-5,6-epoxide**	21.33	19.99	21.77	21.74	15.43	18.40	13.64	17.18	21.57	**19.0**	**3.0**	**15.9**
**Pentadecane**	2.36	2.79	1.65	1.86	1.32	1.68	1.65	1.57	1.82	**1.9**	**0.4**	**24.1**
**Dihydro-actinidiolide**	11.49	9.75	9.91	9.82	6.96	8.77	7.76	8.51	11.23	**9.4**	**1.5**	**16.1**
**Hexadecane**	7.04	6.56	6.00	6.79	5.50	5.85	5.24	5.97	6.26	**6.1**	**0.6**	**9.6**
**Heptadecane**	11.15	12.65	11.58	10.01	10.11	9.43	6.70	10.48	10.25	**10.3**	**1.6**	**16.1**
**Octadecane**	4.16	5.85	4.10	4.39	5.36	3.08	2.57	4.45	3.22	**4.1**	**1.1**	**25.7**
**Farnesyl acetone**	4.91	5.28	7.71	7.50	5.20	5.23	3.97	5.78	3.29	**5.4**	**1.4**	**26.6**
**Eicosane**	0.54	1.09	0.35	1.02	1.10	0.39	0.46	0.74	0.49	**0.7**	**0.3**	**45.0**
**Heneicosane**	0.25	0.25	0.07	0.62	0.55	0.21	0.42	0.59	0.39	**0.4**	**0.2**	**51.1**
**Hexadecanoic acid, butyl ester**	7.88	4.12	22.58	16.68	17.23	9.84	14.78	13.78	7.16	**12.7**	**5.9**	**46.3**
**Docosane**	0.17	0.23	0.11	0.62	0.50	0.20	0.52	0.62	0.41	**0.4**	**0.2**	**53.4**
**Tricosane**	0.08	0.13	0.06	0.63	0.52	0.26	0.49	0.68	0.46	**0.4**	**0.2**	**65.1**

**Sample**	Infected_Char	Infected_Char	Infected_Char	Infected_Char	Infected_Char	Infected_Char	Infected_Char	Infected_Char	Infected_Char	**Mean Conc. (ng/g)**	**Stdev**	**%RSD**
**GC/MS Batch Sample ID**	7	8	9	22	23	24	36	37	38			
**Mass (g)**	0.5012	0.5135	0.4995	0.5007	0.5002	0.5009	0.4035	0.499	0.5045			
**Hexanal**	323.9	225.3	260.1	167.2	154.1	189.5	222.4	176.0	261.1	**220.0**	**54.9**	**25.0**
**2-Hexenal, (E)-**	1304	843.9	1030	1194	1015	839.0	1487	1247	1186	**1127.3**	**214.5**	**19.0**
**5-Hepten-2-one, 6-methyl-**	144.7	135.4	151.6	81.0	125.5	97.7	160.5	103.4	82.6	**120.3**	**30.0**	**25.0**
**o-Cymene**	3.1	2.2	2.3	2.1	2.3	2.9	4.3	3.3	3.5	**2.9**	**0.7**	**25.9**
**1-Hexanol, 2-ethyl-**	10.4	6.5	5.8	12.0	9.0	7.4	12.6	10.2	6.3	**8.9**	**2.5**	**28.6**
**Benzyl alcohol**	28.0	18.5	18.0	50.3	55.4	51.1	44.9	31.1	16.6	**34.9**	**15.7**	**45.0**
**p-Cymenene**	10.3	9.3	11.3	8.5	6.8	15.6	18.9	18.6	21.1	**13.4**	**5.2**	**39.1**
**Phenylethyl Alcohol**	26.6	30.3	30.2	37.6	32.3	34.7	33.3	37.2	31.6	**32.7**	**3.5**	**10.8**
**2,6-Nonadienal, (E,Z)-**	16.3	14.0	15.5	12.0	14.6	17.8	18.6	21.6	28.6	**17.7**	**5.0**	**28.2**
**Methyl salicylate**	2.67	0.54	1.43	1.41	0.98	0.97	4.16	0.93	0.29	**1.5**	**1.2**	**81.5**
**Decanal**	19.32	9.95	14.56	17.99	8.67	12.88	32.12	20.04	15.61	**16.8**	**7.0**	**41.5**
**1-Cyclohexene-1-carboxaldehyde, 2,6,6-trimethyl-**	3.68	3.13	2.81	2.24	2.22	2.29	3.80	3.91	4.54	**3.2**	**0.8**	**26.6**
**ß-Citral**	4.40	1.35	1.19	1.56	0.83	1.18	1.79	1.03	1.29	**1.6**	**1.1**	**66.3**
**α-Citral**	12.14	5.32	4.84	5.31	4.32	5.10	5.79	5.60	3.94	**5.8**	**2.4**	**42.0**
**Propanoic acid, 2-methyl-, 3-hydroxy-2,2,4-trimethylpentyl ester**	110.6	79.38	95.16	97.37	53.47	95.12	130.7	127.6	111.3	**100.1**	**23.9**	**23.9**
**Tetradecane**	2.43	2.08	1.78	2.43	0.68	1.70	1.61	1.27	1.09	**1.7**	**0.6**	**35.5**
**α-Ionone**	3.99	2.89	2.90	1.90	1.28	1.48	3.18	2.89	3.04	**2.6**	**0.9**	**33.7**
**α,ß-Dihydropseudoionone**	34.26	32.77	36.92	28.49	27.63	29.54	36.44	31.4	29.58	**31.9**	**3.4**	**10.7**
**9-epi-ß-Caryophyllene**	2.64	3.43	3.49	3.67	0.88	2.09	1.45	1.19	0.84	**2.2**	**1.2**	**53.0**
**trans-ß-Ionone**	6.81	4.28	4.62	3.77	2.31	2.99	5.04	6.12	6.27	**4.7**	**1.5**	**32.6**
**ß-Ionon-5,6-epoxide**	20.58	16.11	15.30	13.84	9.23	11.81	19.88	20.68	20.25	**16.4**	**4.2**	**25.8**
**Pentadecane**	1.87	1.80	1.87	1.59	0.42	1.11	1.16	1.16	0.84	**1.3**	**0.5**	**38.5**
**Dihydro-actinidiolide**	7.45	7.08	7.29	6.23	5.51	6.17	8.19	9.13	10.27	**7.5**	**1.5**	**20.2**
**Hexadecane**	7.36	5.44	6.55	4.90	1.66	3.96	6.01	5.36	3.64	**5.0**	**1.7**	**34.3**
**Heptadecane**	13.24	9.15	11.34	7.66	3.30	7.14	11.39	8.61	5.93	**8.6**	**3.1**	**35.5**
**Octadecane**	5.73	2.94	3.81	2.34	1.75	3.10	6.29	4.80	3.43	**3.8**	**1.5**	**40.2**
**Farnesyl acetone**	7.04	5.71	6.80	3.97	4.04	5.55	4.55	4.52	4.66	**5.2**	**1.1**	**21.9**
**Eicosane**	0.67	0.35	0.30	0.30	0.31	0.51	1.17	0.76	0.77	**0.6**	**0.3**	**52.2**
**Heneicosane**	0.24	0.04	0.11	0.12	0.11	0.23	0.57	0.60	0.98	**0.3**	**0.3**	**94.7**
**Hexadecanoic acid, butyl ester**	5.76	17.04	14.01	11.88	14.71	9.33	18.32	7.97	8.60	**12.0**	**4.3**	**36.3**
**Docosane**	0.13	0.03	0.02	0.07	0.09	0.07	0.53	0.44	0.42	**0.2**	**0.2**	**100.6**
**Tricosane**	0.01	0.00	0.00	0.13	0.13	0.16	0.58	0.54	0.54	**0.2**	**0.2**	**106.6**

Sample	Char	Char	Char	Char	Char	Char	Char	Char	Char	Mean Conc. (ng/g)	Stdev	%RSD
**GC/MS Batch Sample ID**	10	11	12	16	17	18	30	31	32			
**Mass (g)**	0.5024	0.5026	0.5017	0.4992	0.5003	0.5015	0.5019	0.5016	0.5034			
**Hexanal**	200.1	147.0	194.4	200.2	163.5	240.0	128.8	182.4	321.7	**197.6**	**56.9**	**28.8**
**2-Hexenal, (E)-**	1162.4	968.4	924.6	1065.1	1147.4	842.0	921.4	1313.6	1004.5	**1038.8**	**147.9**	**14.2**
**5-Hepten-2-one, 6-methyl-**	165.0	150.3	110.3	125.1	124.7	80.6	140.9	173.7	94.2	**129.4**	**31.3**	**24.2**
**o-Cymene**	2.2	1.6	2.5	1.7	2.3	2.5	2.2	2.1	2.7	**2.2**	**0.4**	**16.6**
**1-Hexanol, 2-ethyl-**	6.7	6.4	7.1	4.7	8.4	6.2	5.2	6.1	6.2	**6.3**	**1.0**	**16.5**
**Benzyl alcohol**	21.7	62.9	17.4	65.9	55.1	22.3	27.3	63.1	21.4	**39.7**	**21.3**	**53.6**
**p-Cymenene**	10.0	7.2	11.3	7.5	12.1	10.9	13.6	9.4	12.4	**10.5**	**2.2**	**20.8**
**Phenylethyl Alcohol**	31.0	38.3	33.7	33.9	36.6	28.9	33.9	34.9	30.6	**33.5**	**3.0**	**8.9**
**2,6-Nonadienal, (E,Z)-**	25.2	14.6	14.3	14.1	18.7	14.5	15.0	18.4	14.3	**16.6**	**3.7**	**22.3**
**Methyl salicylate**	0.39	0.48	0.98	1.40	0.46	0.50	1.37	2.19	1.15	**1.0**	**0.6**	**60.9**
**Decanal**	13.22	15.01	10.51	33.23	14.71	15.39	24.83	14.44	21.38	**18.1**	**7.1**	**39.5**
**1-Cyclohexene-1-carboxaldehyde, 2,6,6-trimethyl-**	4.98	2.81	3.12	2.34	3.26	2.49	1.92	2.78	2.48	**2.9**	**0.9**	**30.1**
**ß-Citral**	1.40	1.19	1.59	1.02	1.62	1.27	1.17	1.15	1.42	**1.3**	**0.2**	**15.8**
**α-Citral**	5.93	5.58	6.29	4.19	6.79	4.31	5.57	4.58	6.07	**5.5**	**0.9**	**16.8**
**Propanoic acid, 2-methyl-, 3-hydroxy-2,2,4-trimethylpentyl ester**	100.7	59.42	85.64	92.28	100.4	103.4	120.9	63.41	110.1	**92.9**	**20.5**	**22.0**
**Tetradecane**	2.01	1.72	2.13	2.12	2.81	2.53	2.57	1.29	2.09	**2.1**	**0.5**	**21.5**
**α-Ionone**	4.76	2.31	3.32	3.06	2.74	2.85	2.10	2.81	2.34	**2.9**	**0.8**	**27.1**
**α,ß-Dihydropseudoionone**	38.56	34.26	33.58	35.01	32.59	27.99	32.66	35.43	28.86	**33.2**	**3.3**	**9.8**
**9-epi-ß-Caryophyllene**	3.75	3.07	4.25	2.66	3.92	2.35	2.49	0.91	1.75	**2.8**	**1.1**	**38.6**
**trans-ß-Ionone**	8.38	3.99	5.14	4.44	4.97	4.07	3.95	4.16	4.09	**4.8**	**1.4**	**29.4**
**ß-Ionon-5,6-epoxide**	28.57	13.89	18.85	15.22	18.51	13.72	12.72	14.82	15.50	**16.9**	**4.9**	**28.8**
**Pentadecane**	2.01	1.66	1.93	2.02	2.06	1.58	2.07	0.82	1.68	**1.8**	**0.4**	**22.8**
**Dihydro-actinidiolide**	12.98	7.56	8.82	7.51	8.45	6.42	6.09	7.95	6.88	**8.1**	**2.0**	**25.3**
**Hexadecane**	7.10	5.04	6.10	5.95	6.40	5.79	7.22	3.40	5.87	**5.9**	**1.1**	**19.5**
**Heptadecane**	14.16	6.92	10.75	11.04	8.85	1.39	12.71	6.45	10.54	**9.2**	**3.8**	**41.8**
**Octadecane**	5.26	2.71	3.20	3.91	3.78	4.47	4.68	3.79	4.06	**4.0**	**0.8**	**19.2**
**Farnesyl acetone**	8.59	6.73	6.21	6.08	5.13	3.40	3.76	6.45	3.39	**5.5**	**1.8**	**31.9**
**Eicosane**	0.60	0.24	0.16	1.56	1.12	0.99	1.90	1.38	0.90	**1.0**	**0.6**	**59.4**
**Heneicosane**	0.29	0.03	0.00	1.57	0.91	0.74	2.17	1.35	0.94	**0.9**	**0.7**	**81.8**
**Hexadecanoic acid, butyl ester**	8.80	6.11	11.33	20.98	15.00	12.27	17.45	9.27	7.93	**12.1**	**4.9**	**40.0**
**Docosane**	0.04	0.00	0.00	1.58	0.99	0.76	2.46	1.59	1.07	**0.9**	**0.8**	**89.8**
**Tricosane**	0.06	0.00	0.01	1.08	0.73	0.55	1.90	1.35	1.04	**0.7**	**0.7**	**88.5**
