# Supplementary material for: The Effect of Scale Insects on Growth Parameters of cv. Chardonnay and cv. Sauvignon Blanc Grapevines Grown in a Greenhouse
Source: Int J Mol Sci. 2023 Jan 12;24(2):1544. doi: 10.3390/ijms24021544 (PMC9866550; doi:10.3390/ijms24021544)
Supplement: Supplementary file 1 [file ijms-24-01544-s001.zip › TableS1_Updated.pdf]

Table S1. Volatile organic compounds (VOCs) that were found by HS-SPME-GC/MS. RT=retention time, CAS# = chemical abstracts service registry number, NIST = National Institute of Standards and Technology (mass spectral library), FM = mass spectral forward match, RM = mass spectral reverse match, RI = Kovats non-isothermal retention index, PubChem CID = compound identification number, MSI = Metabolomics Standard Initiative. Calculated RI refers to the RT of each endogenous VOC respective to the RTs of the *n*-alkanes in the 10 µg/L of n-hydrocarbon standard mix (C_9–C_{22}) during the GC/MS analysis and calculated based on the Van den Dool and Kratz RT conversion equation for non-isothermal Kovats RI and matched to the RI values found in the NIST and PubChem databases.

Name	Biochemical class	RT (min)	Formula	Base Peak (m/z)	CAS #	NIST FM	NIST RM	NIST RI	Calculated RI	PubChem RI	PubChem CID	Authentic Standards	MSI confidence level
Pentanoic acid	Acid	8.069	C5H10O2	60.1	109-52-4	768	830	903±17 (48)	892	872	7991	Yes	1
3-Heptenoic acid	Acid	13.767	C7H12O2	68.1	29901-85-7	720	733	1081	1082		5282710		2
Octanoic acid	Acid	16.631	C8H16O2	60.1	124-07-2	780	857	1180±7 (100)	1178	1178	379		2
Nonanoic acid *	Acid	19.432	C9H18O2	60.1	112-05-0	871	927	1273±7 (96)	1277	1277	8158		2
Ethanol, 2-butoxy-	Alcohol	8.413	C6H14O2	57.1	111-76-2	772	869	906±2 (22)	905	905	8133		2
1-Octen-3-ol	Alcohol	10.692	C8H16O	57.1	3391-86-4	766	927	980±2 (355)	981	981	18827		2
3-Ethyl-4-methylpentan-1-ol *	Alcohol	11.970	C8H18O	69.1	38514-13-5	874	936	1023±3 (3)	1023	1023	549664		2
1-Hexanol, 2-ethyl-	Alcohol	12.192	C8H18O	57.1	104-76-7	760	877	1030±3 (90)	1031	1031	7720	Yes	1
Phenylethyl Alcohol	Alcohol	14.823	C8H10O	91	60-12-8	927	940	1116±5 (261)	1117	1117	6054	Yes	1
1-Nonanol *	Alcohol	16.483	C9H20O	56.1	143-08-8	901	933	1173±2 (62)	1173	1173	8914		2
Ethanol, 2-(2-butoxyethoxy)-	Alcohol	16.913	C8H18O3	45.1	112-34-5	881	918	1192±4 (15)	1188	1188	8177		2
2-Propanol, 1-(2-butoxy-1-methylethoxy)- (isomer 1)	Alcohol	18.378	C10H22O3	59.1	29911-28-2	842	875		1239		24752		3
2-Propanol, 1-(2-butoxy-1-methylethoxy)- (isomer 2)	Alcohol	18.513	C10H22O3	59.1	29911-28-2	842	875		1244		24752		3
Propanoic acid, 2-methyl-, 3-hydroxy-2,2,4-trimethylpentyl ester	Alcohol	22.084	C12H24O3	71.1	77-68-9	856	878	1380±0 (2)	1374	1380	6490	Yes	1

1-Dodecanol	Alcohol	24.740	C12H26O	55.1	112-53-8	858	951	1473±4 (64)	1474	1474	8193		2
2-Pentenal, (E)-	Aldehyde	4.850	C5H8O	55.1	1576-87-0	809	834	754±5 (30)	764	724	5364752		2
Hexanal	Aldehyde	5.749	C6H12O	56	66-25-1	831	893	800±2 (453)	800	755	6184	Yes	1
2-Hexenal, (E)-	Aldehyde	7.100	C6H10O	41	6728-26-3	885	915	854±3 (243)	853	800	5281168	Yes	1
2,4-Hexadienal, (E,E)-	Aldehyde	8.677	C6H8O	81.1	142-83-6	906	942	911±3 (32)	913	913	637564		2
2-Heptenal, (Z)-	Aldehyde	10.018	C7H12O	41.1	57266-86-1	864	955	958±6 (19)	958	958	5362616		2
(E)-4-Oxohex-2-enal	Aldehyde	10.163	C6H8O2	83.1	2492-43-5	836	859	958±N/A (1)	963	976	6365145		2
2,4-Heptadienal, (Z,Z)-	Aldehyde	11.277	C7H10O	81		849	889		1001	1001	6429263		3
Octanal	Aldehyde	11.422	C8H16O	43.1	124-13-0	819	931	1003±2 (364)	1005	1005	454	Yes	1
2,4-Heptadienal, (E,E)-	Aldehyde	11.713	C7H10O	81	4313-03-5	875	880	1012±4 (108)	1015	1015	5283321		2
2-Octenal, (E)-	Aldehyde	13.116	C8H14O	55	2548-87-0	854	870	1060±3 (124)	1061	1061	5283324		2
Nonanal	Aldehyde	14.534	C9H18O	41	124-19-6	846	850	1104±2 (556)	1108	1108	31289		2
2,6-Nonadienal, (E,Z)-	Aldehyde	15.961	C9H14O	41.1	557-48-2	902	924	1155±3 (124)	1156	1156	643731	Yes	1
2-Nonenal, (E)-	Aldehyde	16.173	C9H16O	43.1	18829-56-6	851	914	1162±3 (223)	1163	1163	5283335		2
2-Nonenal, 8-oxo-	Aldehyde	17.349	C9H14O2	43	2658-60-8	759	783		1203		250594		3
Decanal	Aldehyde	17.504	C10H20O	43.1	112-31-2	887	911	1206±2 (406)	1208	1208	8175	Yes	1
2,4-Nonadienal	Aldehyde	17.819	C9H14O	81.1	6750-03-4	909	954	1213±10 (32)	1219	1219	5283339		2
Undecanal	Aldehyde	20.353	C11H22O	43	112-44-7	830	855	1307±2 (152)	1310	1310	8186		2
5-Hepten-2-one, 6-methyl-	Apocarotenoid	10.847	C8H14O	43	110-93-0	887	930	986±2 (222)	986	986	9862	Yes	1
1-Cyclohexene-1-carboxaldehyde, 2,6,6-trimethyl-*	Apocarotenoid	17.958	C10H16O	137.1	432-25-7	879	909	1220±3 (75)	1224	1224	9895	Yes	1
β-Citral	Apocarotenoid	18.443	C10H16O	41.1	5392-40-5	929	929	1240±3 (168)	1241	1241	643779	Yes	1
α-Citral	Apocarotenoid	19.274	C10H16O	69.1	141-27-5	860	875	1270±2 (36)	1271	1271	638011	Yes	1
alpha.-Ionone	Apocarotenoid	23.413	C13H20O	121.1	127-41-3	849	853	1426±4 (69)	1424	1424	5282108	Yes	1
Dihydropseudoionone	Apocarotenoid	24.010	C13H22O	43	689-67-8	927	931	1456±5 (14)	1447	1447	1549778	Yes	1
trans-.beta.-Ionone	Apocarotenoid	24.909	C13H20O	177.1	79-77-6	913	918	1486±4 (210)	1481	1481	638014	Yes	1
β-Ionon-5,6-epoxide	Apocarotenoid	25.003	C13H20O2	123.1	23267-57-4	913	925	1473±18 (9)	1484	1484	5352481	Yes	1

3-Oxo-α-ionol *	Apocarotenoid	28.649	$C_{13}H_{20}O_2$	108.1	34318-21-3	876	911	1647±16 (13)	1651		273524347		2
Farnesylacetone (5E,9E)	Apocarotenoid	31.553	$C_{18}H_{30}O$	43	1117-52-8	901	906	1919±5 (28)	1915	1915	1711945	Yes	1
Benzaldehyde	Benzenoid	10.228	C_7H_6O	77.1	100-52-7	800	919	962±3 (416)	965	965	240	Yes	1
Benzyl alcohol *	Benzenoid	12.439	C_7H_8O	79.1	100-51-6	876	919	1036±4 (174)	1039	1039	244	Yes	1
Benzeneacetaldehyde	Benzenoid	12.724	C_8H_8O	91.1	122-78-1	801	904	1045±4 (378)	1048	1048	998		2
Phenol, 2-methoxy-3-(2-propenyl)-*	Benzenoid	21.611	$C_{10}H_{12}O_2$	164.1	25586-57-6	792	808	1377±15 (2)	1357	1362	596373		2
Benzofuran, 5-methoxy-6,7-dimethyl-	Benzenoid	24.599	$C_{11}H_{12}O_2$	161.1	35355-35-2	755	859		1469		595346		3
1,8(2H,5H)-Naphthalenedione, hexahydro-8a-methyl-, cis-	Benzenoid	26.085	$C_{11}H_{16}O_2$	124.1		730	753		1527		580218		3
Hexadecanoic acid, butyl ester	Ester	33.509	$C_{20}H_{40}O_2$	56.1	111-06-8	793	847	2188±N/A (1)	2187	2188	8090	Yes	1
Tetradecane	Hydrocarbon	22.769	$C_{14}H_{30}$	57.1	629-59-4	922	928	1400	1400	1400	12389	Yes	1
Pentadecane	Hydrocarbon	25.426	$C_{15}H_{32}$	57.1	629-62-9	881	886	1500	1500	1500	12391	Yes	1
Hexadecane	Hydrocarbon	27.891	$C_{16}H_{34}$	57.1	544-76-3	886	879	1600	1600	1600	11006	Yes	1
Heptadecane	Hydrocarbon	29.387	$C_{17}H_{36}$	57	629-78-7	805	810	1700	1700	1700	12398	Yes	1
Octadecane	Hydrocarbon	30.509	$C_{18}H_{38}$	57.1	593-45-3	847	854	1800	1800	1800	11635	Yes	1
Eicosane	Hydrocarbon	32.225	$C_{20}H_{42}$	57	112-95-8	891	895	2000	2000	2000	8222	Yes	1
Heneicosane *	Hydrocarbon	32.937	$C_{21}H_{44}$	57	629-94-7	850	854	2100	2100	2100	12403	Yes	1
Docosane *	Hydrocarbon	33.591	$C_{22}H_{46}$	57	629-97-0	884	885	2200	2200	2200	12405	Yes	1
Tricosane	Hydrocarbon	34.202	$C_{23}H_{48}$	57	638-67-5	910	911	2300	2300	2300	12534	Yes	1
2-Pentanone, 4-hydroxy-4-methyl-	Ketone	6.696	$C_6H_{12}O_2$	43.1	123-42-2	808	908	838±8 (27)	837	784	31256		2
2,5-Furandione, 3,4-dimethyl-	Ketone	12.328	$C_6H_6O_3$	54.1	766-39-2	876	908	1038±N/A (1)	1035	1038	13010		2
1H-Pyrrole-2,5-dione, 3-ethyl-4-methyl-	Nitrogen volatile	18.320	$C_7H_9NO_2$	139.1	20189-42-8	865	936	1239±4 (7)	1237	1238	29995		2
1H-Pyrrole-2,5-dione, 3-ethenyl-4-methyl-	Nitrogen volatile	19.106	$C_7H_7NO_2$	66.1	21494-90-6	727	831	1261±N/A (1)	1265	1261	152426		2

Oxepine, 2,7-dimethyl-	Other	9.271	C8H10O	122.1	1487-99-6	805	829	944±10 (2)	933	934	578868		2
2H-Pyran-2,6(3H)-dione *	Other	11.168	C5H4O3	55	5926-95-4	883	948		997		574367		2
3,5-Octadien-2-one, (E,E)-	Oxylipin	13.453	C8H12O	95.1	30086-02-3	841	889	1073±7 (13)	1072	1072	5352876		2
Methyl salicylate	Oxylipin	17.185	C8H8O3	120.1	119-36-8	775	916	1192±2 (145)	1197	1197	4133	Yes	1
Benzothiazole	Sulfur volatile	18.198	C7H5NS	135.1	95-16-9	831	917	1229±8 (60)	1233	1234	7222		2
Cyclohexene, 3-(1-methylethyl)- *	Terpenoid	9.599	C9H16	81	3983-08-2	785	802	924±3 (7)	944	948	520966		2
o-Cymene	Terpenoid	12.075	C10H14	119.1	527-84-4	945	957	1022±2 (116)	1027	1027	10703	Yes	1
2,6-Octadiene, 2,6-dimethyl-	Terpenoid	12.899	C10H18	69.1	2792-39-4	817	844	978±1 (27)	1054	990	5365898		2
p-Cymenene	Terpenoid	14.106	C10H12	117.1	1195-32-0	885	938	1090±2 (106)	1093	1093	62385	Yes	1
Geraniol *	Terpenoid	18.767	C10H18O	69.1	106-24-1	825	846	1255±3 (343)	1253	1253	637566		2
Cyclopropanemethanol, 2-methyl-2-(4-methyl-3-pentenyl)-	Terpenoid	20.646	C11H20O	69.1	98678-70-7	806	809		1321		549579		3
Geranyl vinyl ether	Terpenoid	20.837	C12H20O	69.1		747	811		1328		5365842		3
Cyclohexene, 1,5,5-trimethyl-6-acetylmethyl-	Terpenoid	20.934	C12H20O	123	211563-96-1	817	817		1331		579163		3
beta.-Gurjunene	Terpenoid	23.694	C15H24	161.1	17334-55-3	805	896	1432±3 (234)	1435		28481		2
9-epi-β-Caryophyllene	Terpenoid	24.452	C15H24	91.1		927	963	1466±3 (24)	1463	1460	6429301	Yes	1
β-Selinene	Terpenoid	25.269	C15H24	105.1	17066-67-0	872	920	1486±3 (349)	1494	1494	442393		2
α-Selinene	Terpenoid	25.473	C15H24	189	473-13-2	860	894	1494±3 (196)	1502		10856614		2
γ-Cadinene	Terpenoid	25.969	C15H24	161.1	39029-41-9	853	921	1513±2 (485)	1522	1515	92313		2
6,10-Dodecadien-1-yn-3-ol, 3,7,11-trimethyl-	Terpenoid	26.181	C15H24O	69.1	2387-68-0	782	797	1562±N/A (1)	1530.629747	1562	5365823		2
cis-Calamenene	Terpenoid	26.228	C15H22	159.1	72937-55-4	726	785	1531±N/A (1)	1533	1567	6429077		2
Dihydroactinidiolide	Terpenoid	26.508	C11H16O2	111.1	17092-92-1	949	954	1532±10 (26)	1544	1537	27209	Yes	1

Epiglobulol	Terpenoid	27.275	C15H26O	43.1		828	861		1575		11858788		2
(-)-Spathulenol	Terpenoid	27.579	C15H24O	43	77171-55-2	882	894	1577±5 (6)	1587		13854255		2
Globulol *	Terpenoid	27.775	C15H26O	43.1	489-41-8	806	865	1583±3 (203)	1595	1590	12304985		2
Spiro[4.5]decan-7-one, 1,8-dimethyl-8,9-epoxy-4-isopropyl-*	Terpenoid	27.937	C15H24O2	43.1		781	792	1626	1603		538938		2
Phytol *	Terpenoid	33.035	C20H40O	71.1	150-86-7	804	882	2114±5 (66)	2115	2115	5280435		2
